# Of Tracts, Rings, Nodes, Cusps, Sinuses, and Arrhythmias—A Comment on Szili-Torok *et al.*’s Paper Entitled “The ‘Dead-End Tract’ and Its Role in Arrhythmogenesis”. *J. Cardiovasc. Dev. Dis*. 2016, *3*, 11

**DOI:** 10.3390/jcdd3020017

**Published:** 2016-04-19

**Authors:** Robert H. Anderson, Diane E. Spicer, Shumpei Mori

**Affiliations:** 1 Institute of Genetic Medicine, Newcastle University, Newcastle-upon-Tyne NE1 3BZ, UK; 2 Johns Hopkins All Children’s Heart Institute, St. Petersburg, FL 33701, USA; spicerpath@hotmail.com; 3 Division of Pediatric Cardiology, University of Florida, Gainesville, FL 32608, USA; 4 Division of Cardiovascular Medicine, Department of Internal Medicine, Kobe University Graduate School of Medicine, Kobe 650-0017, Japan; shumpei_8@hotmail.com

**Keywords:** dead-end tract, retroaortic node, conduction tissues

## 1. Introduction

In the review, now published as part of the special issue devoted to the development of the conduction tissues, de Vries and his colleagues discuss the potential role of the so-called “dead-end tract” as a substrate for arrhythmogenesis [1]. One of us was privileged to review their submission during its iterations prior to publication. We are grateful to the authors for including the illustration that we provided showing the location of the tract in the human heart. It is surprising that, in 2016, there should still be uncertainty regarding the full extent of the so-called “conduction tissues”. It is all the more surprising that we still do not know the functions of the lesser-known components, nor whether they justify description as parts of the “conduction system”.

## 2. How Do We Define the “Conduction System”?

It has now been over a century since Tawara first described the atrioventricular conduction axis. His stellar description is now available in an excellent English translation [2]. It is also over one hundred years since Aschoff and Monkeberg outlined excellent criteria for the histological recognition of conducting tracts [3,4]. Their reviews were stimulated by a meeting of the German Society of Anatomy, at which a session had been convened to debate the suggestion made by Thorel, namely that a “tract” of cardiomyocytes was to be found within the atrial walls connecting the sinus node with the atrioventricular conduction axis [5]. The very existence of “specialized” internodal tracts remains a contentious issue today, with multiple images available from Google illustrating purported tracts in a fashion comparable to the ventricular conduction pathways. In their separate reviews, Aschoff and Monkeberg argued that, to be considered an anatomically discrete tract within the walls of the cardiac chambers, cardiomyocytes should be histologically discrete, should be traceable within serial histologic sections, and, most importantly, should be insulated from the adjacent myocardium. For the purposes of distinguishing the gross anatomical features of the components of the “cardiac conduction system”, there is no reason to presume that these criteria have lost their validity. We can now refine the criteria, nonetheless, so as to distinguish between the cardiac nodes and insulated tracts [6]. In this light, when considering “specialized internodal tracts”, it may well prove possible, using immunocytochemical markers, and when examining the developing heart, to identify broad pathways within the atrial walls between the sinuatrial and the atrioventricular junctions. We are unaware, however, of any studies that, in the postnatal heart, have shown pathways extending between the sinus and atrioventricular nodes that fulfill the third criterion of the German giants, namely that the cardiomyocytes within the purported “tract” are insulated from the adjacent myocardium. It is this third criterion that is the key to identifying additional “tracts” within the heart not currently recognized as representing parts of the “conduction system”.

## 3. What, then, Is the “Dead-End Tract”?

**Figure 1:**
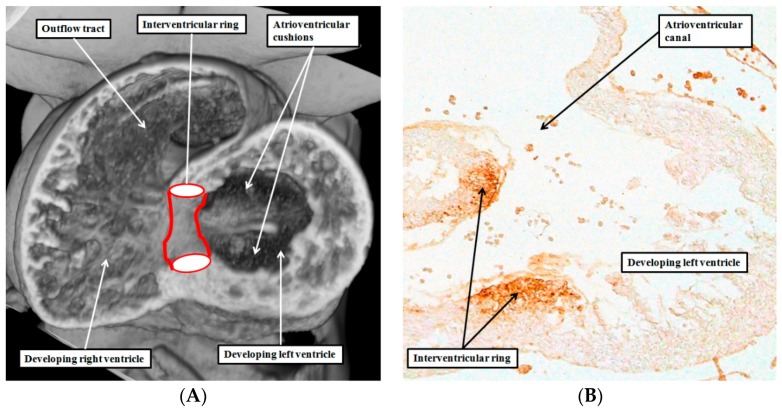
The left hand panel (**A**) is taken from an episcopic dataset prepared from a mouse embryo at E10.5. It is cut along the long axis of the ventricular loop, and shows the location of the interventricular ring, which has the inner heart curvature as its cranial margin, and the developing muscular ventricular septum as the caudal margin; The right hand panel (**B**) is a comparable section from a human embryo processed with an antibody to the nodose ganglion of the chick heart. It shows that the cranial margin of the interventricular ring not only occupies the inner heart curvature, but also forms the rightward margin of the atrioventricular canal. The original section was prepared in Amsterdam by Professor Andy Wessels, and is reproduced with his permission.

The dead-end tract, which is certainly one of these well-recognized components, fulfills the German criteria. It should, therefore, be considered as belonging to the “conduction system”. The studies performed by the Amsterdam group, spearheaded by Wessels, revealed the developmental origin of the tract [7,8]. It is part of a ring of cardiomyocytes demonstrated by their reaction to the nodose ganglion of the chick heart. The ring, when first seen, and not at this stage insulated from the neighboring myocardium, encircles the interventricular communication of the developing heart. At this stage, the atrioventricular canal is supported exclusively by the developing left ventricle, and the outflow tract arises exclusively from the cavity of the developing right ventricle (Figure 1A).

Since the cardiomyocytes retain their reaction to the antibody in subsequent development, the ring becomes molded in the form of a Mobius strip [7]. The molding occurs as the atrioventricular canal expands so as to open into the developing right ventricle, and the proximal outflow tract grows so as to achieve a connection with the developing left ventricle. The end result is that part of the ring of stained cardiomyocytes extends from the newly formed septum to encircle the tricuspid valvar orifice, with the remainder of the ring encircling the aortic root. The course of the remolded interventricular ring is directly comparable to the known distribution of the tracts of cardiomyocytes in the avian heart, these tracts fulfilling the German criteria for recognition as conducting tissues [9,10].

## 4. Are There Other Rings of “Conducting Tissues”?

In mammalian hearts [11,12], and in the developing human heart [13], an immunocytochemically discrete ring of cells is also to be found encircling the entirety of the atrioventricular junctions. A component of this atrioventricular ring, which runs within the vestibule of the tricuspid valve, is confluent with the comparable part of the interventricular ring. This is because, when first seen, the interventricular ring is itself also an atrioventricular structure (Figure 1B). Subsequent to the insulation of the atrial from the ventricular myocardial masses, the cardiomyocytes within the immunocytochemically discrete ring become sequestrated on the atrial side of the fibrous insulating barrier.

## 5. Is There a Difference between “Tracts” and “Nodes”?

Using the criteria suggested by Aschoff [3] and Monkeberg [4], it is now possible to distinguish between “tracts” and “nodes” [6]. A node, if defined literally, is no more than a crossing point, or intersection. When we examine the relationships between the interventricular and the atrioventricular rings, we can recognize two such crossing points. The points are equivalent to the transitions in the avian atrioventricular conduction system where the tracts pass from the atrial to the ventricular myocardial masses. For the mammalian heart, it was Tawara who clarified the location of the structure now known as the atrioventricular node, showing that it formed the proximal extent of the atrioventricular conduction axis. Tawara also showed, in his initial work, the presence of extensions from the node into both the tricuspid and mitral vestibules. The extensions are the remnants of the developmental rings, the node itself being formed at their dorsal intersection. In the mammalian heart, furthermore, the nodal myocytes are no longer insulated from the adjacent working atrial cardiomyocytes. In the developing mammalian hearts, there is then a second intersection between the atrioventricular and interventricular rings. This intersection corresponds with the ventral atrioventricular transition found in the avian hearts. There is then a second node, recognizable using the German criteria, to be found at this site of ventral intersection in mammalian hearts. This is the retroaortic node, which is just as poorly recognized currently as the dead-end tract and, like that tract, as yet has no known function [14].

## 6. Are There Differences between the Developmental Structures and Their Postnatal Remnants?

In the postnatal human heart, when using the German criteria, it is no longer possible to recognize the continuity of the tracts which can be identified in the developing heart using immunocytochemical markers, and which exist in the avian heart as insulated entities. It is possible, nonetheless, to recognize remnants of the histologically specialized cardiomyocytes that fulfill two of the German criteria, namely, that they are discrete in terms of their staining characteristics when compared to their working neighbors, and that they can be traced in serial histological sections. These persisting remnants are the so-called “atrioventricular ring tissues” [14,15]. It is these remnants, and not the dead-end tract, as is suggested by de Vries and colleagues [1], which form part of the circuits for atrioventricular re-entry, specifically for the so-called Mahaim tachycardia [16]. It is also these remnants that were mistakenly described by Kent as forming multiple muscular atrioventricular connections in the normal heart [17]. In the normal heart, it is the atrioventricular conduction axis, developed from the central part of the interventricular ring, which forms the solitary conducting pathway between the atrial and ventricular myocardial masses. As is correctly emphasized by de Vries and his colleagues [1], nonetheless, it is the dead-end tract, and not the right bundle branch, which is the anterior continuation of this axis.

## 7. Are the Remnants the Substrates for Idiopathic Ventricular Tachycardias?

**Figure 2:**
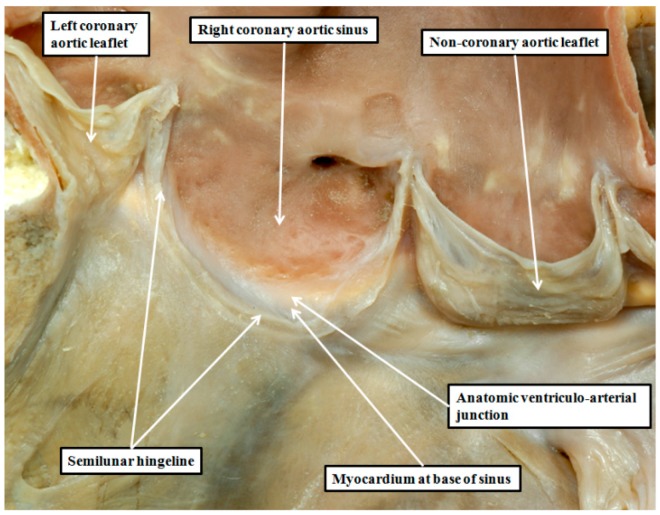
The image shows the aortic root of a normal heart opened by a cut though the left coronary aortic sinus, and viewed from the front. The leaflet guarding the right coronary aortic sinus has been removed, revealing its semilunar line of attachment. The hingeline crosses proximally beyond the anatomic ventriculo-arterial junction, thus incorporating a crescent of myocardium in the base of the sinus.

De Vries and his associates are correct when they suggest that the dead-end tract is a possible substrate for the idiopathic ventricular arrhythmias that can be ablated from the sinuses of the aortic valve [1]. Similar arrhythmias, however, are also known to originate from the right ventricular outflow tract [18], and these can be ablated from the sinuses of the pulmonary valve. It should not be assumed, therefore, that all of the so-called outflow tract arrhythmias have their genesis from the dead-end tract. To understand the alternative substrates for arrythmogenesis, we need to consider in greater detail the anatomy and the relationships of the arterial valvar sinuses. It is unfortunate in this regard that many describe the valvar sinuses using the word “cusp”. This word is also used on occasion to describe the valvar leaflets, not only of the arterial valves, but also of the atrioventricular valves. It is unsuitable for use in either setting. When used in its vernacular sense, the word describes a point or elevation, or the crossing of two curves. Old anatomists presumably employed the word to describe the leaflets of the cardiac valves because of their similarities, when seen in their closed state, to the raised surfaces of the molar and premolar teeth. If we are to take advantage of the points emphasized by de Vries and associates [1], it is now essential to distinguish between the leaflets and the sinuses of the arterial roots. The leaflets are the moving parts, while the sinuses are the walls which support the leaflets in semilunar fashion (Figure 2).

**Figure 3:**
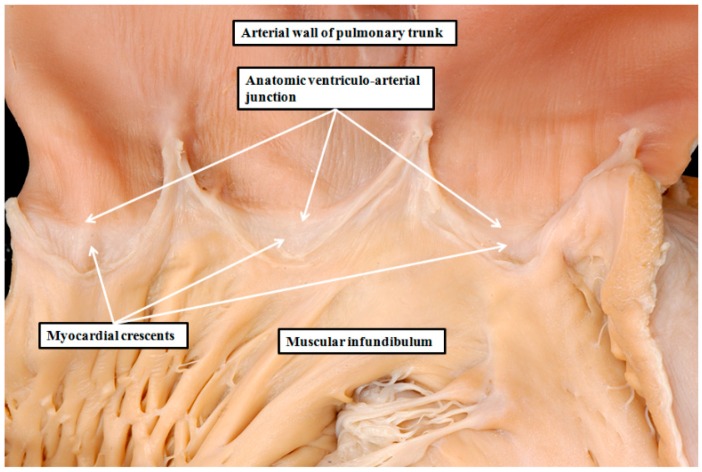
The image shows the right ventriculo-arterial junction of the normal heart. The heart has been opened by making a cut through the parietal component of the junction. The leaflets of the pulmonary valve have been removed to reveal their semilunar lines of attachment. This makes it possible also to recognize the circular anatomic ventriculo-arterial junction formed at the boundary between the muscular right ventricular infundibulum and the arterial wall of the pulmonary trunk. Because the hingelines of all three leaflets cross the anatomic ventriculo-arterial junction, there are crescents of infundibular myocardium incorporated at the bases of each of the pulmonary valvar sinuses. It is these myocardial components that are ablated when abolishing right ventricular outflow tract tachycardias from the pulmonary trunk.

Distinguishing between leaflets and sinuses in this fashion permits not only an understanding of the location of the dead-end tract, but also the complex anatomic arrangement of the valvar sinuses, and the relations between the aortic and pulmonary roots. The larger parts of the valvar sinuses, in both the aortic and pulmonary roots, have walls composed of arterial tissues. The boundary between the myocardial walls of the ventricles and the arterial walls is the anatomic venticulo-arterial junction, best seen in the pulmonary root, where it forms a complete circle (Figure 3). The anatomic junction, however, does not correspond with the hemodynamic ventriculo-arterial junction. The latter junction is provided by the semilunar hingelines of the arterial valvar leaflets. In the pulmonary root, each of these hingelines crosses the anatomic ventriculo-arterial junction, thus incorporating crescents of myocardium into the base of the three sinuses (Figure 3). It is almost certain that these myocardial components of the pulmonary valvar sinuses harbor the substrates for right ventricular outflow tract tachycardias, and which are ablated from the sinuses [19]. 

## 8. Are There Differences in the Anatomy of the Arterial Roots?

**Figure 4:**
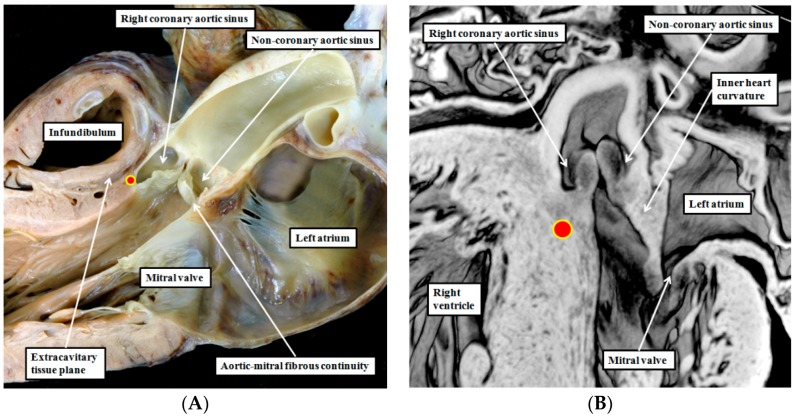
The left hand panel (**A**) shows the outflow tract of the normal left ventricle sectioned to replicate the so-called parasternal long axis echocardiographic plane. It shows the fibrous continuity between the aortic leaflet of the mitral valve and the non-coronary leaflet of the aortic valve. The red circle with yellow borders shows the location of the dead-end tract. Note the extracavitary tissue plane between the crest of the ventricular septum and the subpulmonary infundibulum; The right hand panel (**B**) is taken from an episcopic dataset prepared from a developing mouse embryo at E15.5. By this stage, the embryonic interventricular communication is closed, but the inner heart curvature forming the roof of the left ventricle, and interposing between the developing leaflets of the aortic and mitral valves, remains myocardial. The red circle with yellow borders again shows the location of the dead-end tract.

The uniform arrangement as found in the pulmonary root is not to be seen in the aortic root, where only two of the sinuses have myocardial support. The dorsal part of the aortic root is made up of fibrous continuity between the leaflets of the aortic and mitral valves (Figure 4A). The fibrous continuity is not present when the aortic root initially achieves its connection with the developing left ventricle. At the initial time of closure of the embryonic interventricular communication, the inner heart curvature forming the roof of the left ventricle remains myocardial (Figure 4B).

Only with ongoing development are the myocardial walls converted into fibrous tissue. The two sinuses of the aortic root giving rise to the coronary arteries, however, retain their myocardial support. The support of the right coronary aortic sinus is provided by the crest of the muscular ventricular septum. This is the location of the dead-end tract, wedged between the septal myocardium and the arterial wall of the right coronary aortic sinus. As the tract extends into the left coronary aortic sinus, it moves away from the septum. In this location, the myocardium supporting the sinusal wall is part of the wall of the primary heart tube, which is transferred into the left ventricle along with the aortic root. As primary, rather than chamber, myocardium, it too could be arrhythmogenic [19].

## 9. Can We Demonstrate the Specific Anatomy during Life?

**Figure 5:**
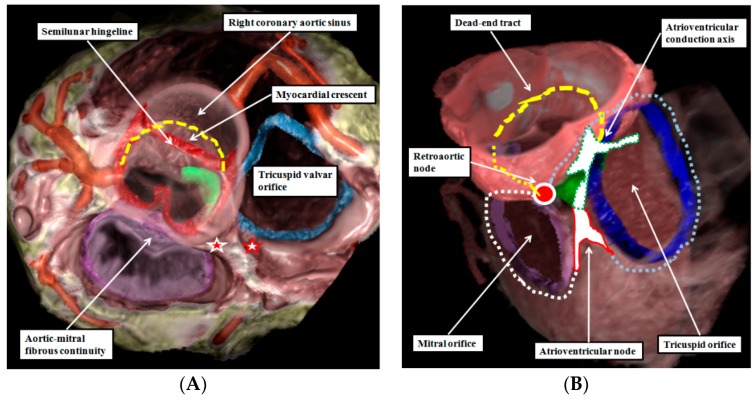
The images are taken from a dataset prepared from a patient undergoing investigation for coronary arterial disease using multi-detector computed tomography. The dataset has been reconstructed to show the semilunar hingelines of the aortic valve in red, the annulus of the tricuspid valve in blue, and the location of the membranous septum in green. The reconstruction is viewed from the cranial aspect in the left hand panel (**A**), with the left side of the heart seen to the left hand of the observer. The location of the dead-end tract is marked by the yellow dashed line. The white star with red borders shows the location of the atrioventricular node, while the red star with white borders shows the location of the retroaortic node; The right hand panel (**B**) shows the same reconstruction viewed obliquely from above and from the right. In this view, the location of the atrioventricular node is shown by the while area with red borders, with the atrioventricular conduction axis shown as the white area with dotted green borders. The tricuspid ring is shown by the blue dotted line, and the mitral ring by the yellow dotted line. The dead-end tract is again shown by the yellow dashed line, with the “missing” part of the initial interventricular ring in the area of aortic-to-mitral fibrous continuity shown by the dotted yellow line. The view of the reconstruction in the right hand panel shows how the atrioventricular node and the retroaortic node, shown by the red circle with white borders, are formed at the intersections of the atrioventricular and interventricular rings.

The extent of the myocardial components of the aortic sinuses, and the location of the dead-end tract, can now be demonstrated in the living heart by interrogation of datasets obtained using multi-detector computed tomography (Figure 5).

The reconstructions show that it is most unlikely that the dead-end tract is responsible for the arrhythmias originating from the outflow tract of the right ventricle. This is because the free-standing infundibular myocardial sleeve can now be shown to lift the pulmonary root away from the base of the ventricular mass. The computed tomographic datasets show the extracavitary fibrous tissue that interposes between the crest of the muscular ventricular septum, and hence the site of the dead-end tract, and the bases of the pulmonary valvar sinuses. The pulmonary root, of course, can be liberated from the heart, and used by the surgeon as an autograft in the Ross procedure [20]. This very fact points to the lack of any “outlet muscular septum” in the postnatal heart.

## 10. Conclusions

De Vries and his colleagues concluded their own review with the suggestion that “more efficient targeting of this possible origin of idiopathic ventricular arrhythmias could help to further improve ablation outcomes” [1]. We endorse their suggestion. As we now show, clinicians have the techniques to locate the site of the dead-end tract with exquisite accuracy at their fingertips. In future, we can anticipate that they will determine the sites of their ablations with equal accuracy, testing directly the hypotheses advanced by de Vries and colleagues [1].
